# Cerebral Metastases from Malignant Fibrous Histiocytoma of Bone

**DOI:** 10.1080/13577140020008093

**Published:** 2000-09

**Authors:** Susanne J. Rogers, Jeremy S. Whelan

**Affiliations:** London Bone and Soft Tissue Tumour Service Meyerstein Institute of Oncology Middlesex Hospital Mortimer Street London W1N 8AA UK

## Abstract

Four patients with malignant fibrous histiocytoma of bone (MFH-B) metastasizing to brain are reported. In two cases, signs of cerebral involvement developed between 4 and 28 months after diagnosis. Both patients had known pulmonary or bony metastases. As a consequence of this experience, two further patients were subsequently identified, one with a definite cerebral metastasis and one who had an asymptomatic supratentorial lesion, possibly metastatic. It is suggested that patients with MFH-B and widespread metastatic disease at presentation or developing within a short interval should undergo cerebral imaging.


Sarcoma (2000) **4**, 125–128

CASE REPORT

### Cerebral metastases from malignant fibrous histiocytoma of bone

SUSANNE J. ROGERS & JEREMY S. WHELAN

*London Bone and Soft Tissue Tumour Service, Middlesex Hospital, London, UK*

**Abstract**
Four patients with malignant fibrous histiocytoma of bone (MFH-B) metastasizing to brain are reported. In two cases, signs of cerebral involvement developed between 4 and 28 months after diagnosis. Both patients had known pulmonary or bony metastases. As a consequence of this experience, two further patients were subsequently identified, one with a definite cerebral metastasis and one who had an asymptomatic supratentorial lesion, possibly metastatic. It is suggested that patients with MFH-B and widespread metastatic disease at presentation or developing within a short interval should undergo cerebral imaging.

**Key words**: *malignant fibrous histiocytoma of bone, brain, cerebral, metastases*

### Introduction

Malignant fibrous histiocytoma arising in bone (MFH-B) is an uncommon but well defined primary spindle cell tumour, predominantly affecting adults.[1] The tendency to affect long bones and metastasize to the lung encourages comparison with osteosarcoma (OS). Similarly, neoadjuvant chemotherapy with the agents active in OS appears to improve survival in MFH-B.[2,3] In this report we describe a previously unreported metastatic site of MFH-B and discuss the implications for the clinical management of this disease.

### Case histories

### *Patient 1*

A 48-year-old female presented with a 5-month history of pain in the left arm. Plain radiographs revealed a predominantly lytic lesion and a pathological fracture of the metaphysis of the proximal humerus. No other abnormalities were demonstrated on staging scans which included isotope bone scan, computed tomography (CT) of the thorax, mammography and abdominal ultrasound. Core needle biopsy diagnosed a tumour with bundles of spindle-shaped fibroblastic cells in a storiform pattern with a high mitotic rate, consistent with MFH-B. Neoadjuvant chemotherapy with doxorubicin and cisplatin was commenced.[3] The first cycle was complicated by febrile neutropenia and acute renal failure, so carboplatin was substituted for cisplatin. Six cycles of chemotherapy were administered, with endoprosthetic replacement of the proximal humerus after the second. Radiotherapy (60 Gy in 30 fractions) completed the treatment. The patient remained well for 11 months before developing pain in the right leg. An isotope bone scan was consistent with metastases in the fibula and also in the 9th rib. Local radiotherapy was given but further metastases were evident on a bone scan 2 months later, and renal and adrenal tumour deposits were seen on abdominal CT.

Three months later, she became agitated and disorientated and demonstrated inappropriate behaviour. Contrast-enhanced CT showed multiple enhancing lesions in both cerebral hemispheres. Further palliative radiotherapy was given, but the patient died 8 weeks later, 30 months after diagnosis.

### *Patient 2*

A 45-year-old male presented with a pathological fracture of the left distal femur, sustained whilst exercising. Lytic areas in the cortex, with subperiosteal new bone formation, were seen adjacent to the fracture site on plain radiography. Magnetic resonance imaging (MRI) revealed a large lesion in the distal metaphysis and core needle biopsy was diagnostic of MFH-B. Isotope bone scan showed this to be unifocal bone disease. However, bilateral pulmonary metastases were evident on CT thorax. Prior to surgery, he received two cycles of ifosfamide (9 g/m$^2$). He failed to attend for post-operative chemotherapy but presented 1 month later with three

Correspondence to: J. Whelan, London Bone and Soft Tissue Tumour Service, Meyerstein Institute of Oncology, Middlesex Hospital, Mortimer Street, London W1N 8AA, UK. Tel: +44 (0) 171380 9092; Fax: +44 (0) 171 436 0160; E-mail: jeremy.whelan@uclh.org

1357-714Xprint/1369-1643online/00/030125-04 © 2000 Taylor & Francis Ltd
*DOI: 10.1080/13577140020008093*

grand mal seizures. CT of the head showed multiple metastases with considerable oedema (Fig. 1A,B). The fits were controlled pharmacologically and palliative whole-brain radiotherapy was given (30 Gy in 10 fractions). He died 7 months after diagnosis.

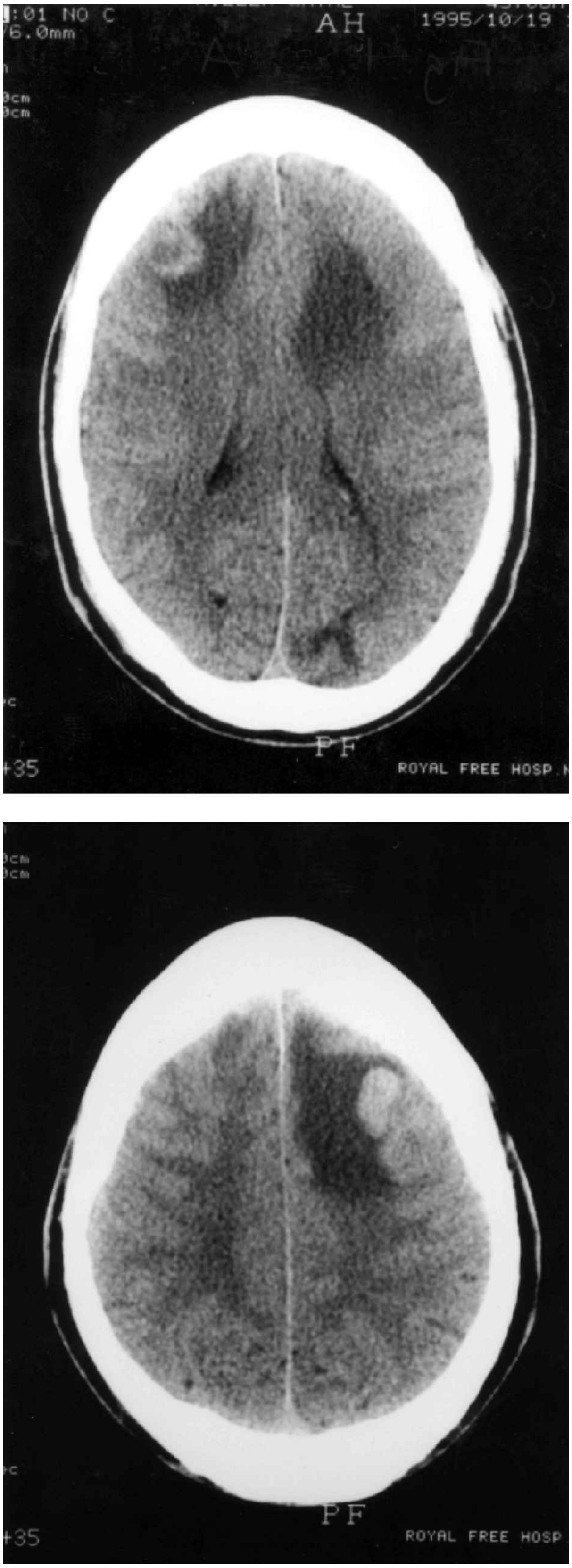

**Fig. 1A,B.** *CT head scan of patient 2, showing multiple cerebral metastases.*

*Patient 3*

A 54-year-old female described an 18-month history of pain around the right knee, exacerbated by weight bearing. CT showed a lesion near the medial condyle of the right femur and histological review of the bone biopsy confirmed MFH-B. Staging CT of the chest and isotope bone scan were negative for metastases. Two cycles of chemotherapy were given pre-operatively (methotrexate 4 g/m$^2$ day 1, ifosfamide 3 g/m$^2$ and doxorubicin 60 mg/m$^2$ day 10) and 2 further cycles were administered after endoprosthetic replacement.

Ten years later, metastases were detected in the left lung on routine chest X-ray and, at thoracotomy, four nodules consistent with MFH-B were resected. She received six cycles of chemotherapy with ifosfamide, doxorubicin and dacarbazine post-operatively. However, a 6 cm skin recurrence overlying the prosthesis was detected 1 year later. This was excised and followed by local radiotherapy (60 Gy in 30 fractions). A CT of the thorax 4 months later revealed progressive disease in the right lung with possible new metastases in the left lung.

In view of the protracted disease course and multiple recurrences, before proceeding to further chemotherapy or surgery, further assessment included a CT scan of the brain. This detected a large asymptomatic supratentorial lesion, of which the differential diagnosis was a meningioma or a metastasis. Biopsy was considered but rejected as unjustifiably dangerous in the presence of no symptoms and disease elsewhere. An MRI scan was performed 3 months later which showed no change in the cerebral lesion (Fig. 2). The patient remains asymptomatic and thoracotomy has been recommended.

*Patient 4*

A 40-year-old man had experienced pain in the right flank for 6 years. The severity had increased in the

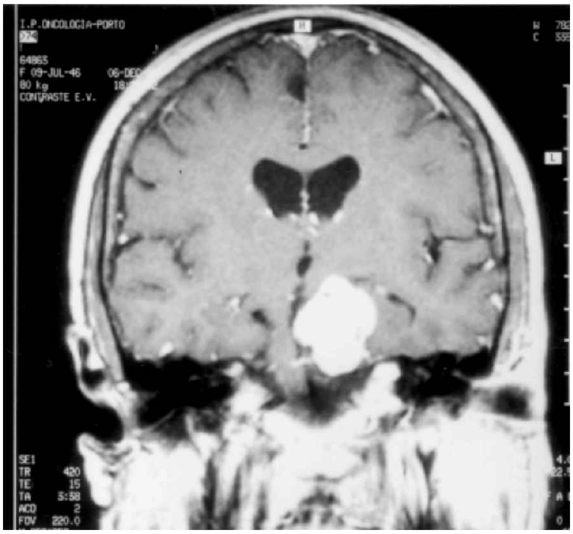

**Fig. 2.** *MRI head scan of patient 3, showing supratentorial mass.*

preceding 6 months, presumed secondary to a groin strain, but had failed to respond to rest and analgesia. A plain X-ray of the pelvis showed a large osteolytic lesion destroying the right acetabulum and ischial spine with a large associated soft-tissue mass. MRI of the pelvis confirmed these findings and bone biopsy revealed MFH-B. Staging CT thorax and abdomen showed multiple bilateral lung metastases, para-aortic lymphadenopathy and a left adrenal metastasis. With consideration given to experience of patients 1–3, a CT head scan was performed and was normal. Palliative chemotherapy was initiated with doxorubicin and cisplatin.[3] Symptomatic improvement following chemotherapy lasted only days and, after the third cycle, palliative radiotherapy (8 Gy in a single fraction) was given to the right hemipelvis. He then noticed frequent twitching around the left corner of the mouth consistent with focal fits. A left upper motor neurone VIIth nerve palsy rapidly developed and he started to vomit. CT head showed a right frontal lesion with associated oedema (Fig. 3). Steroids and anti-convulsants were commenced with resolution of the symptoms. In view of his poor response to chemotherapy and deteriorating condition, he was transferred to a hospice for palliative care and died 3 weeks later, 4 months after diagnosis.

Patient details are summarized in Table 1.

### Discussion

MFH-B represents approximately 5% of primary bone tumours and occurs predominantly in the long bones of patients aged 10–60 years, with a predilection for the distal femur and proximal tibia in 50%.[1] Twenty per cent of MFH-B arises in abnormal bone, e.g. bone infarcts, Paget's disease and enchondroma,[4] or after radiotherapy.[5] MFH-B is differentiated histologically from osteosarcoma by characteristic features which include spindle-shaped fibroblastic cells in a storiform pattern and an absence of osteoid formation.

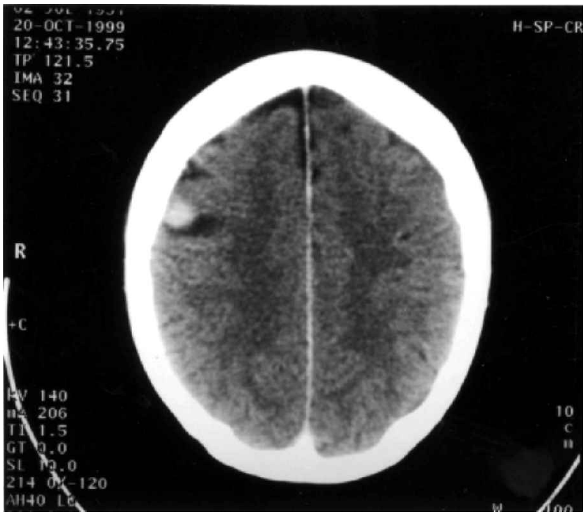

**Fig. 3.** *CT head scan of patient 4, showing cerebral metastasis.*

**Table 1.** *Patient characteristics*

| Patient | Age (years) | Sex | Site of disease at diagnosis: Primary | Site of disease at diagnosis: Metastases | Treatment of primary tumour: Pre-operative | Treatment of primary tumour: Post-operative | Time of relapse (months post-diagnosis) | Site(s) of relapse | Overall survival (months) |
|---|---|---|---|---|---|---|---|---|---|
| 1 | 48 | F | Left humerus | Nil | 1×dox/cisplatin<br>1×dox/carboplatin | 4×dox/carboplatin | 19<br>26<br>28 | Bone<br>Adrenal<br>Brain | 30 |
| 2 | 45 | M | Left femur | Right and left lungs | 2×ifosfamide | Refused | 4 | Brain | 7 |
| 3 | 54 | F | Right distal femur | Nil | 2×methotrexate/ ifosfamide/dox | 2×methotrexate/ ifosfamide/dox | 120<br>138<br>148 | Lung<br>Skin and lung<br>Brain? | Alive at 153 months |
| 4 | 40 | M | Right hemipelvis | Right and left lungs<br>Para-aortic lymph nodes (LN) and adrenal | 1×dox/cisplatin<br>1×dox/cisplatin 50%<br>8 Gy in 1 fraction | No surgery<br>Palliative care | 3 | Brain | 4 |

Historically, treatment with surgery or radiotherapy alone resulted in disease recurrence in 70% of patients within 5 years.[6] The two main sites of early metastases are lung and bone. A series of 76 cases reported the development of metastases in 52 patients. Forty-four had pulmonary involvement, 6 had bony spread and 1 had positive lymph nodes.[6] Renal, hepatic, adrenal, cardiac, peritoneal, cutaneous and pleural lesions have also been described.[4,7] The brain is recognized as an unusual site of metastasis in patients with osteosarcoma (1.5% incidence at autopsy).[8] Craniotomy is a treatment option, although the diagnosis of cerebral metastases is often a terminal event. There is a single case report of 8-year disease-free survival in a child treated with chemotherapy alone for bilateral cerebral metastases from an osteosarcoma.[9] Retrospective analysis of 254 cases of osteosarcoma yielded 13 who had disease recurrence (median 3 months after diagnosis) and subsequently developed cerebral metastases (median 10 months after relapse).[9] The patients mostly had pulmonary or bony involvement at presentation and particularly aggressive disease behaviour. It has been suggested that patients with osteosarcoma with metastatic disease at or within 12 months of diagnosis should have CT or MRI of the brain.[9]

We have now reported cerebral metastases in MFH-B. Imaging of the brain is required for all patients with symptoms or signs suggesting cerebral involvement. The similarities in the disease behaviour between osteosarcoma and MFH-B lead us to recommend that brain imaging should be considered in patients with widespread pulmonary and/or bone metastases at relapse, particularly if the disease-free interval is short. It remains to be seen whether early detection of cerebral metastases from MFH-B improves survival.

### Acknowledgements

We would like to thank Mr J. Cobb for granting us permission to report patient 1, and Mr S. Cannon for patients 2, 3 and 4.

### References

1 Mirra JM. *Bone tumours*. Philadelphia: Lea & Felbiger, 1989;771–80.

2 Earl H, Pringle J, Kemp H, Morittu L, Miles D, Souhami R. Chemotherapy of malignant fibrous histiocytoma of bone. *Ann Oncol* 1993;4:409–15.

3 Bramwell V, Steward W, Nooij M, Whelan J, Craft A, Grimer R *et al.* Neoadjuvant chemotherapy with doxorubicin and cisplatin in malignant fibrous histiocytoma of bone: a European Osteosarcoma Intergroup Study. *J Clin Oncol* 1999;17:3260–9.

4 Spanier S. Malignant fibrous histiocytoma of bone. *Orthop Clin N Am* 1997;8:947–61.

5 Dahlin D, Unni K, Matsuno T. Malignant fibrous histiocytoma of bone—fact or fancy? *Cancer* 1977;39:1508–16.

6 Capanna R, Bertoni F, Bacchini P, Bacci G, Guerra A, Campanacci M. Malignant fibrous histiocytoma of bone: the experience at the Rizzoli Institute: report of 90 cases. *Cancer* 1984;54:177–87.

7 Spanier S, Enneking W, Enriquez P. Primary malignant fibrous histiocytoma of bone. *Cancer* 1975;36:2084–98.

8 Danziger J, Wallace S, Handel S, Alonso de Santos L. Metastatic osteogenic sarcoma to the brain. *Cancer* 1979;79:707–10.

9 Marina N, Pratt C, Shema S, Brooks T, Rao B, Meyer W. Brain metastases in osteosarcoma. Report of a long term survivor and review of the St Jude's Children's Hospital experience. *Cancer* 1993;71:3656–60.
